# Linking rare and common disease: mapping clinical disease-phenotypes to ontologies in therapeutic target validation

**DOI:** 10.1186/s13326-016-0051-7

**Published:** 2016-03-23

**Authors:** Sirarat Sarntivijai, Drashtti Vasant, Simon Jupp, Gary Saunders, A. Patrícia Bento, Daniel Gonzalez, Joanna Betts, Samiul Hasan, Gautier Koscielny, Ian Dunham, Helen Parkinson, James Malone

**Affiliations:** European Bioinformatics Institute (EMBL-EBI), European Molecular Biology Laboratory, Wellcome Trust Genome Campus, Hinxton, Cambridge, CB10 1SD UK; Centre for Therapeutic Target Validation, Wellcome Trust Genome Campus, Hinxton, Cambridge, CB10 1SD UK; GSK, Medicine Research Centre, Stevenage, SG1 2NY UK

**Keywords:** Rare disease, Phenotype disease associations, OBAN, CTTV, EFO

## Abstract

**Background:**

The Centre for Therapeutic Target Validation (CTTV - https://www.targetvalidation.org/) was established to generate therapeutic target evidence from genome-scale experiments and analyses. CTTV aims to support the validity of therapeutic targets by integrating existing and newly-generated data. Data integration has been achieved in some resources by mapping metadata such as disease and phenotypes to the Experimental Factor Ontology (EFO). Additionally, the relationship between ontology descriptions of rare and common diseases and their phenotypes can offer insights into shared biological mechanisms and potential drug targets. Ontologies are not ideal for representing the *sometimes associated* type relationship required. This work addresses two challenges; annotation of diverse big data, and representation of complex, *sometimes associated* relationships between concepts.

**Methods:**

Semantic mapping uses a combination of custom scripting, our annotation tool ‘Zooma’, and expert curation. Disease-phenotype associations were generated using literature mining on Europe PubMed Central abstracts, which were manually verified by experts for validity. Representation of the disease-phenotype association was achieved by the Ontology of Biomedical AssociatioN (OBAN), a generic association representation model. OBAN represents associations between a subject and object *i.e.,* disease and its associated phenotypes and the source of evidence for that association. The indirect disease-to-disease associations are exposed through shared phenotypes. This was applied to the use case of linking rare to common diseases at the CTTV.

**Results:**

EFO yields an average of over 80 % of mapping coverage in all data sources. A 42 % precision is obtained from the manual verification of the text-mined disease-phenotype associations. This results in 1452 and 2810 disease-phenotype pairs for IBD and autoimmune disease and contributes towards 11,338 rare diseases associations (merged with existing published work [Am J Hum Genet 97:111-24, 2015]). An OBAN result file is downloadable at http://sourceforge.net/p/efo/code/HEAD/tree/trunk/src/efoassociations/. Twenty common diseases are linked to 85 rare diseases by shared phenotypes. A generalizable OBAN model for association representation is presented in this study.

**Conclusions:**

Here we present solutions to large-scale annotation-ontology mapping in the CTTV knowledge base, a process for disease-phenotype mining, and propose a generic association model, ‘OBAN’, as a means to integrate disease using shared phenotypes.

**Availability:**

EFO is released monthly and available for download at http://www.ebi.ac.uk/efo/.

**Electronic supplementary material:**

The online version of this article (doi:10.1186/s13326-016-0051-7) contains supplementary material, which is available to authorized users.

## Introduction

Drug discovery research involves diverse analytical activities and integration of many sources of data about diverse entities from single nucleotide polymorphisms (SNPs) to pathways, proteins to populations. The Centre for Therapeutic Target Validation (CTTV) is a collaboration between the European Bioinformatics Institute (EMBL-EBI), GlaxoSmithKline (GSK) and the Wellcome Trust Sanger Institute (WTSI) to develop a knowledge base of evidence for drug targets based on genomic experiments and bioinformatics analyses. A CTTV goal is to develop a better understanding of the rare and common disease relationship via shared phenotypes, genes, and pathways, as information from rare disease can provide mechanistic insight to common disease and vice versa. This requires integration of data generated by CTTV projects with existing data residing in EMBL-EBI, WTSI and GSK resources. Data types include variants, genes, proteins, gene expression, pathways, compounds, literature and related experimental variables such as disease and phenotype with data generation on different experimental platforms such as Genome Wide Association Studies and next generation sequencing.

The integration of disease and phenotypic information, where a group of phenotypes are associated with a disease, becomes increasingly important when considering rare diseases where research is typically fragmented across omics types and disease. Rare disease data are not always compatible with each other as they come from different resources, e.g., OMIM [1] and ORPHANET [2], represent different perspectives of the diseases, such as diagnostics or treatment, and data are typically population, or even individual, specific. The sparseness and heterogeneity of this data therefore introduces a major challenge in the integration of rare and common disease information [3].

**Fig. 1:**
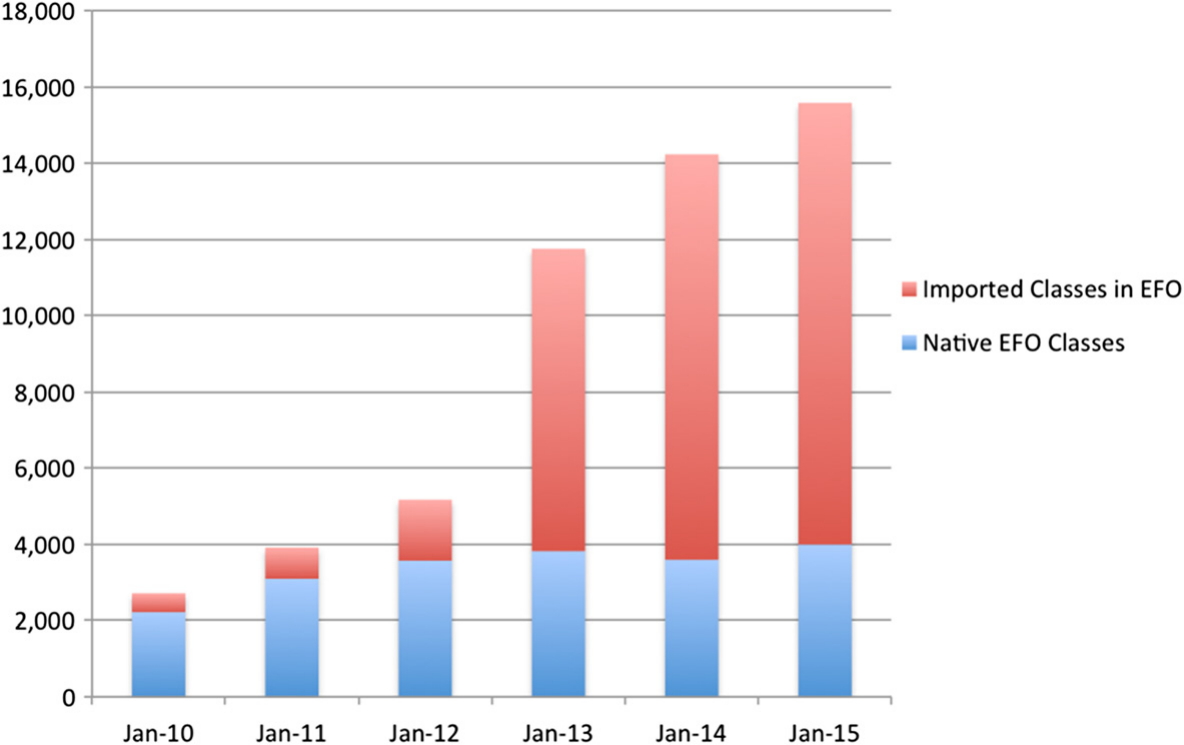
There were 2214 EFO-native classes in January 2010, and 3992 EFO-native classes in January 2015. Although EFO has significantly grown in its number of native classes, the number of imported classes has grown at a much higher rate. Importing more than 6000 rare disease classes from ORDO in 2012, and axiomatizing them into EFO has resulted in a sudden increase between 2012 and 2013. This reflects the use of EFO as an application ontology providing interoperability across domain ontologies through semantic axiomatization

**Fig. 2:**
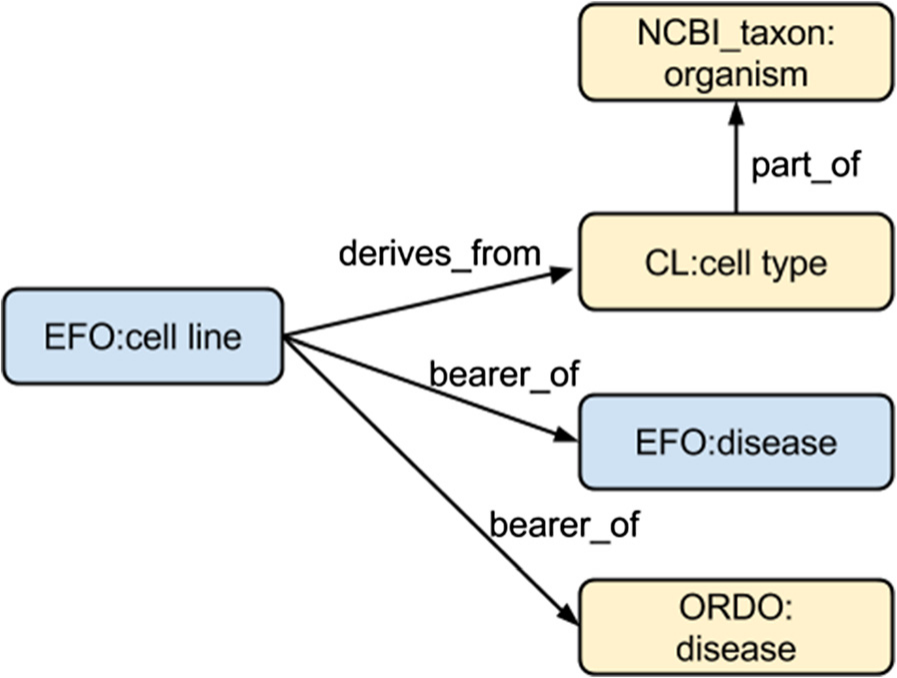
The cell line design pattern in EFO links an EFO class ‘cell line’ to external ontologies via import mechanism. An EFO cell line *derives_from* a cell type class from Cell Ontology, which is *part_of* an organism – a class imported from NCBI Taxon. EFO cell line class is also a *bearer_of* a disease – a class imported from ORDO or class native to EFO itself

CTTV uses the Experimental Factor Ontology (EFO) [4] as its application ontology to provide an integrated and consistent ontological representation of the CTTV platform data. EFO provides an integration framework for ontologies and reuses components of domain-specific ontologies such as Orphanet Rare Disease Ontology (ORDO) [5], ChEBI [6], Gene Ontology [7] and Uberon [8]. Typically a data or use case driven ‘SLIM’ (a subset of the referenced ontology with MIREOT import closures [9]) of a source ontology is created, and then imported into EFO. Figure 1 illustrates the exponential growth of EFO where a large amount of classes are imported from externally-sourced ontologies. This presents challenges representing the imported knowledge in EFO without losing the structural integrity of the original ontologies. We therefore use MIREOT to import classes, or small sections of hierarchies from external ontologies to avoid potentially importing the whole or most of a source ontology into EFO due to the complexity of class organization. This also helps ensure amenability of EFO to wider data integration. For example, rare disease terms are imported from ORDO and phenotypes from Human Phenotype Ontology terms as both ontologies are compatible with EFO’s disease and phenotype design pattern respectively and common disease terms are defined locally with EFO-namespace URI. Even though other ontologies exist that aim to describe disease, there is not one single-origin representation of common disease in any of the available ontologies that is compatible with the current design pattern of disease representation used in EFO, thus creating common disease classes in the EFO namespace is currently necessary for CTTV. Figure 1 shows that despite considerable growth in EFO-native classes (3992 EFO-native classes in 2015, as opposed to 2214 classes in 2010), EFO use of imported classes from external domain ontologies is increasing. EFO uses common design patterns that are consistent throughout the EFO ontology development process (e.g., term creation, and term importing) to integrate and organize the ontologies imported. For example, the design pattern for cell line representation: cell line *derives_from* a cell type, which is *part_of* an organism, which is a *bearer_of* some disease links an EFO’s cell line class to the Cell Ontology’s cell type class, an NCBI Taxonomy class, and EFO’s or ORDO’s disease class. This cell line design pattern as shown in Fig. 2 is also shared with the Cell Line Ontology [10]. Webulous [11] (extended publication in JBMS Bioontologies SIG Thematic issue), a tool which implements these design patterns in a Google Sheets add-on, is used to create new terms (the ‘class’), and to allow users to define new terms for EFO in spreadsheet format. These are transformed to OWL and imported prior to each monthly release. The use of design patterns also provides consistency with other ontology consuming resources such as the EBI RDF Platform [12]. In order to be interoperable with OBO foundry ontologies EFO uses BFO 1.1 [13] upper level classes. For example EFO represents disease as a child of BFO:Disposition [14] whereas, following the same process, HP:phenotype is modelled as a child of BFO:Quality. In EFO, a common design pattern is such that an EFO:disease *has_phenotype* HP: links EFO disease terms and HP. EFO diseases are organized utilizing an object property *has_disease_location* using anatomical classes imported from UBERON.

Data resources integrated into CTTV have local standards for annotation and many aggregate data from multiple external sources, where each external resource also has a resource specific annotation and/or curation process. They have also historically used different ontologies and dictionaries for disease and phenotype annotation; examples include Online Mendelian Inheritance in Man (OMIM) [15], the Systematized Nomenclature of Medicine – Clinical Terms (SNOMED-CT) [16], the Human Disease Ontology (DO) [17], and the Medical Dictionary for Regulatory Activities (MedDRA) [18] as seen in Table 1. We note that these resources often do not differentiate between disease and phenotype when selecting and applying the vocabularies to their data. We have standardized this for CTTV, differentiating phenotype from disease, and defaulting to HP imported terms in EFO for the description of phenotypes where possible. For example, the GWAS Catalog trait myopia is annotated to the HP’s IRI http://purl.obolibrary.org/obo/HP_0000545 ‘Myopia’. EFO therefore contains phenotypic terms that are clearly distinguished from disease terms for annotation of CTTV data.

**Table 1:** An overview of ontologies usage by each CTTV data source. Cross-reference sources of each CTTV data resource are normalized to EFO for CTTV data validation process

Database	Cross-reference annotation sources
EVA	OMIM, SNOMED-CT, MeSH
ArrayExpress	GO, OMIM, EFO
UniProt	OMIM, Orphanet, MeSH
Reactome	OMIM, GO
ChEMBL	MedDRA, ATC, GO
GWAS Catalog	EFO, DO

Diseases are associated with phenotypes which manifest in the disease with qualifying information about the nature of the association. The disease-phenotype association is established to represent disease connections via shared phenotypes. For example, the rare disease Aicardi-Gourtieres syndrome has several associated phenotypes affecting the brain, immune system, and skin, such as microcephaly, hepatosplenomegaly, elevated blood liver enzymes, thrombocytopenia, and abnormal neurological response. It is often not observable at birth, and all phenotypes are unlikely to be present in all patient presentations. Additionally phenotypes may also vary by kindred and/or by population in their frequency and penetrance. The same is true for common disease, for example, phenotypes of Crohn’s disease may range from inflammation of any part of the gut (but most likely ileum or colon), diarrhea, or constipation, but not all symptoms are necessarily present in one patient. Representation of the disease-phenotype association in an OWL ontology with the statement ‘disease *has_phenotype* some phenotype’ requires that all instances of a disease have that specific phenotype and our examples above illustrate that this representation is problematic for many cases. We have therefore chosen to represent disease-phenotype association in a generic association model ‘OBAN’ (the Open Biomedical AssociatioN), which allows us to represent both the disease-phenotype association and qualify the association with evidence, and, in the future, to represent information such as frequency of association. In order to test this model, and to populate it with disease-phenotype associations for Inflammatory Bowel Disease we used a text mining approach to extract these from the literature, building a corpus using an expert nominated set of journals as our experience described in Vasant et al. [19], indicates that constraining the corpus improves precision on post-hoc validation by experts. Abstracts were accessed using the EuropePMC API [20] and the Whatizit text mining pipeline [21] was usd to mine the corpus using a dictionary comprised of phenotype terms from the Human Phenotype Ontology [22] and the Mammalian Phenotype ontology [23].

## Methods

### Mapping CTTV data sources disease and phenotype terms to EFO

In order to perform semantic integration of multiple resources for CTTV, the data from each source (listed in Table 1) was mapped to EFO identifiers. Challenges in performing such mapping pertain in the non-standardized use of vocabulary sets by different resources. Some of the resources used an ontology, e.g., Disease Ontology, a taxonomy such as MeSH [24], or cross-referenced another resource such as OMIM. Diseases and phenotypes are often mixed in the same resource and sometimes in the same category annotation. For example, the European Variation Archive (EVA – http://www.ebi.ac.uk/eva/) [25] trait names’ labeling uses a mixed set of vocabularies from HP, SNOMED-CT, OMIM, and non-standardized local identifiers used internally at source from the ClinVar records. The identifiers of the record’s cross-references for each trait name are not equivalently represented - e.g., trait name ‘congenital adrenal hyperplasia’ in EVA contains identifiers for SNOMED-CT, HP, but not for OMIM. This trait name also links to a non-standardized internal identifier used at the Office of Rare Disease. Another example instance of EVA trait name ‘Epstein syndrome’ only contains a cross-reference to a SNOMED-CT identifier (but not OMIM, nor HP), and a non-standardized internal identifier from Genetic Alliance, a submitter of ClinVar [25]. In EFO, disease classes are cross-referenced to multiple ontologies and vocabularies such as the National Cancer Institute Thesaurus (NCIt) [26], MeSH, OMIM, Anatomical Therapeutic Chemical (ATC) classification [27], or UMLS [28] via the specific *definition_citation* annotation property. These *definition_citation* properties are refined in EFO to indicate the specific vocabulary where the term is cross-referenced from, e.g., OMIM_definition_citation, SNOMEDCT_definition_citation, etc. When importing from external ontologies, additional cross-reference information is absorbed into EFO from the OBOinOWL property *hasDbXref*, such as those used in HP*.* To conform with EFO’s mechanism of *definition_citation*, EFO developers have further added these imported *hasDbXref* annotation values to the corresponding source-specific *definition_citation* for better conformance and coverage when mapping terms by cross-reference links using EFO customized programming script.

To map CTTV terms to EFO, we exploited EFO’s cross-references and mapped identifiers supplied for ontology terms where these were provided and where mappings were 1:1. For example, in UniProt, the human protein Catalase http://www.uniprot.org/uniprot/P04040 is annotated with OMIM:115500 *acatalasia*. EFO contains a cross-reference via *OMIM_definition_citation* for EFO_0004144 Acatalasia. This allows us to then map directly from EFO to Catalase from CTTV via the OMIM ID. We were able to quickly identify and map classes for resources which used some semantic identifiers transparently, even when these were not from an ontology but a resource such as OMIM. In the case of 1-to-many mappings, we programmatically identified the exact match of synonyms in the cross-reference list, and avoided broader or narrower synonyms. However, in other cases, resources such as EVA do not use any semantic identifiers locally and aggregate data from multiple sources that often contain only textual descriptions of diseases and phenotypes. We therefore applied manual curation where a standardized URI was not provided to the data, to carefully map the disease or phenotype annotation. This process was used in addition to the manual curation process used to assign disease terms when the record was initially curated, and serves to harmonize the data. It also includes examination of OMIM entries, and Orphanet data (http://www.orpha.net) to identify mappings that reference genetic and rare diseases where disease and phenotype labeling is not standardized for consistency across multiple databases. This step was coupled with literature review to ensure the accuracy of the mapping. For example, the EVA phenotype term ‘Glucose-6-phosphate transport defect’ was manually mapped to ‘Glycogen storage disease due to glucose-6-phosphatase deficiency type b’ in Orphanet. Non-exact mappings were allowed for the purpose of data integration, provided that mappings were supported by evidence from peer-reviewed literature. Table 2 summarises the coverage of CTTV data mapping to EFO in this study. If a term cannot be mapped to existing terms in EFO, external ontologies are examined for (potential) new terms to import. Failing this, an EFO class is added, and asserted into an appropriate place in the class hierarchy. EFO first attempts to create terms by requesting these from the authoritative reference ontology, for example request of new rare disease term, synonym or cross-reference from ORDO. This avoids generating an EFO term when the scope of work is covered by a reference ontology. Occasionally EFO temporarily creates the term and later imports a term from the reference ontology if and when it becomes available, to avoid delays in data releases. Failing all this, a new EFO class is created under EFO namespace.

**Table 2:** Summary of mapping between textual data annotations and EFO or ORDO ontology classes, following process outlined in methods section (%)

Database	% Annotated to EFO or ORDO
EVA (inc. ClinVar)	89 % of annotations of frequency > 100
ArrayExpress	77 %
UniProt	78 %
Reactome	100 %
ChEMBL	99 %
GWAS Catalog	100 %

### Text mining for candidate disease-phenotype associations

To generate the disease-phenotype association knowledge base for Inflammatory Bowel Disease (IBD) and autoimmunity disorders, a two-step process was performed in this pipeline. First, a corpus was identified using the European PubMed Central web services [29]. SOAP web services were used to download all abstracts from journal articles that were annotated with the diseases that were subclasses of Inflammatory Bowel Disease in EFO, their preferred label (for example ‘Crohn’s disease’) and all their MeSH synonyms (for example, granulomatous colitis, Crohn’s granulitis, etc). In order to mine for the co-occurrence of disease and phenotype terms Whatizit [21], a dictionary-based text mining tool was used. A dictionary composed of terms from the Human Phenotype ontology (HP) and the Mammalian Phenotype ontology (MP) was then used as the reference for phenotype terms. This dictionary was used as input to the Europe PMC hosted *Whatizit* pipeline, which was applied to the abstracts identified in the first stage. This process returned a list of candidate disease-phenotype associations formatted as a spreadsheet containing columns for Term Frequency, Inverse Document Frequency, associated phenotype terms and abstract links (please follow the links in Additional file 1 and Additional file 2). EBI curators performed initial cleaning of nonspecific terms – for example the HP contains the terms ‘All’, ‘Chronic’, or ‘death’. Three GSK clinicians then reviewed and verified the true positive candidate associations before the final list of disease-phenotype associations was transformed into OWL format corresponding to OBAN as described below.

### Building an IBD disease-phenotype association knowledge base with OBAN

A challenge in modeling disease and phenotype connections in an ontological framework is that they are typically considered a ‘sometimes associated’ relationship. Ontologies expressed in OWL are not well suited to describe such relationships because when a property is asserted at the class level, it is interpreted as true at all times [30, 31] and for all members of that class. Therefore, an OWL implementation with a probability value attached to the object property relation between two classes to describe this ‘sometimes-associated’ relation is problematic as the condition would be true for some members of the class. This is particularly problematic when a probability is unknown or constrained, e.g., to a small population sample and support for such constructs is exploratory at best. Exploiting this relationship at the instance level would introduce another ontology modeling complication in EFO, meaning that we would either lose the information at the class level for the information that is always true, or would repeatedly insert that information into every instance of that class. Neither represents a sustainable modeling of such relationship. We have therefore separated the two kinds of relationships. Where connections can be made existentially (the relationship is always true), they are asserted in the ontology as class descriptions via object properties. For example, a disease ‘neoplasm’ is axiomatized in EFO as having the abnormality in the cell proliferation process with a syntax (*realized_in* some (‘disease course’ and ((‘*has part*’ some ‘cell proliferation’) and (*bearer_of* some abnormal)))). This existentially asserts in EFO that a disease class neoplasm is *realized_in* a disease course that bears a quality of some abnormality (*bearer_of* PATO:abnormal) and *has_part* GO Process:’cell proliferation’. When reasoning is performed on EFO, this abnormality of process (i.e., PATO:abnormal of GO:’cell proliferation’) classification is inferred through this asserted axiom clause at class level.

**Fig. 3:**
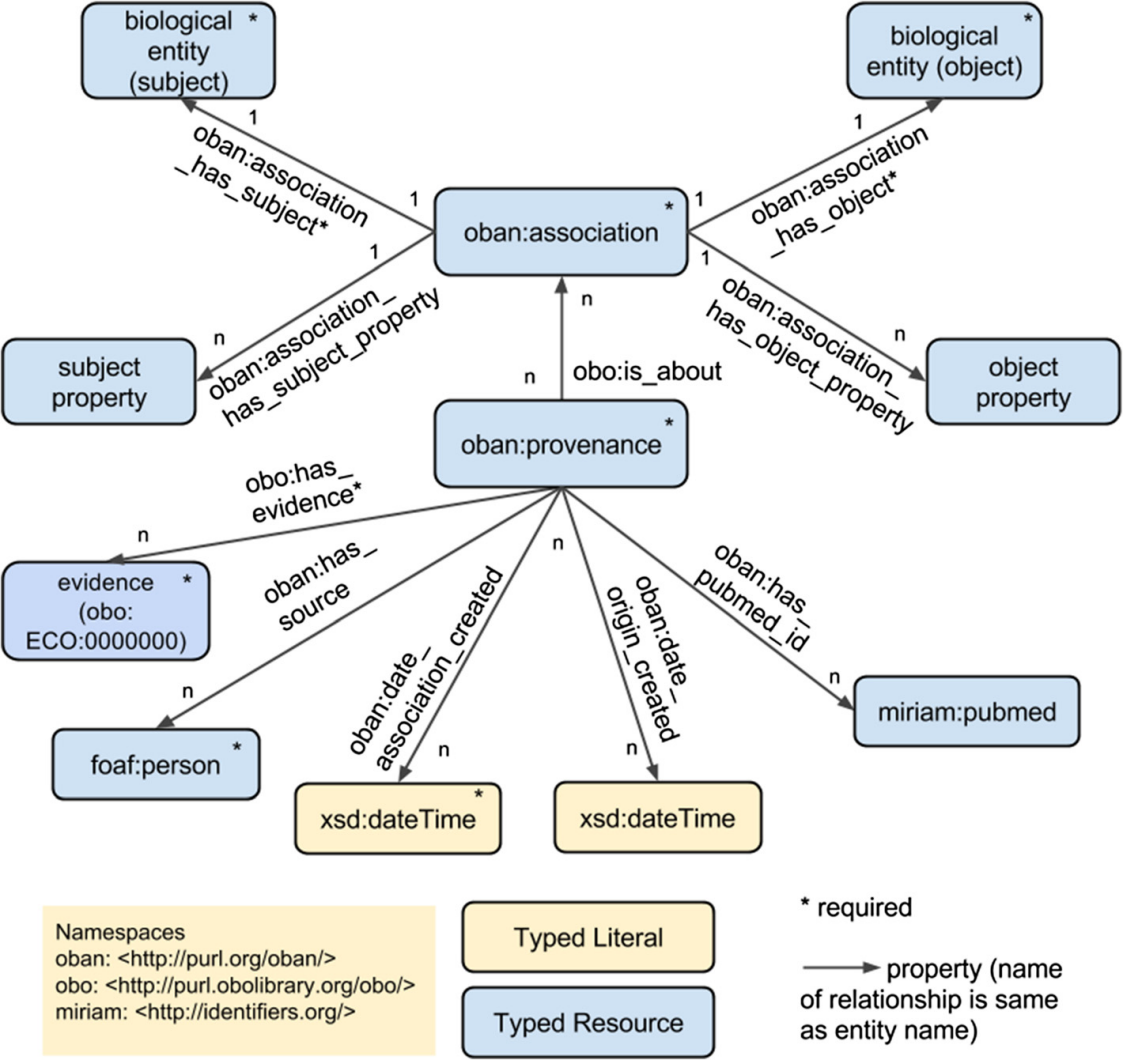
An OBAN association links an entity such as a disease to another such as an associated phenotype and retains the provenance information (e.g., manual curation, published findings, etc). Entities marked with * are required and others are added on per association basis, for instance the PubMed triple in this figure

For other ‘sometimes true’ relationships, the OBAN representation has been designed in an attempt to ease this problem. OBAN (Fig. 3) decouples the relationship between the disease and phenotype classes, and instead makes the relationship about an intermediate class of things – an OBAN association – true for a given disease and a phenotype (conceptualized as two biological entity classes; one represents a subject role, and the other represents an object role in the association). Linkages between a disease and associated phenotype are represented as instances of the class *‘OBAN:association*’, which has one or more ‘*OBAN:provenance*’ instances (see Fig. 3)*.* An association is an OWL class defined in the OBAN ontology (https://github.com/EBISPOT/OBAN) to represent a triple-form entity of subject-related-to object through the object properties *association_has_subject* and *association_has_object*. This association is supported by an *OBAN:provenance* class that instantiates a provenance entity that supports the association. In OBAN, provenance is a class that validates the association statement in the corresponding *OBAN:association* class instance. One provenance individual can also be about several associations as the same paper may provide evidence for multiple disease-phenotype associations, and each association instance can have several items of provenance attached to it. In this work, diseases are typically subjects and phenotypes are modeled as objects but the association is bi-directional *i.e.,* the association class only denotes two entities being associated with each other without enforcing directionality on the link. However, to standardize information within the CTTV, we have elected to customize the use of the OBAN association to have disease as a subject, and phenotype as an object; the subject and object relations are there to enable directionality if required later in the scope of CTTV. For example, an OBAN association is constructed via the syntax *association_has_subject* (EFO:disease) ‘Crohn’s disease’, and *association_has_object* (EFO:phenotype) diarrhea.

**Fig. 4:**
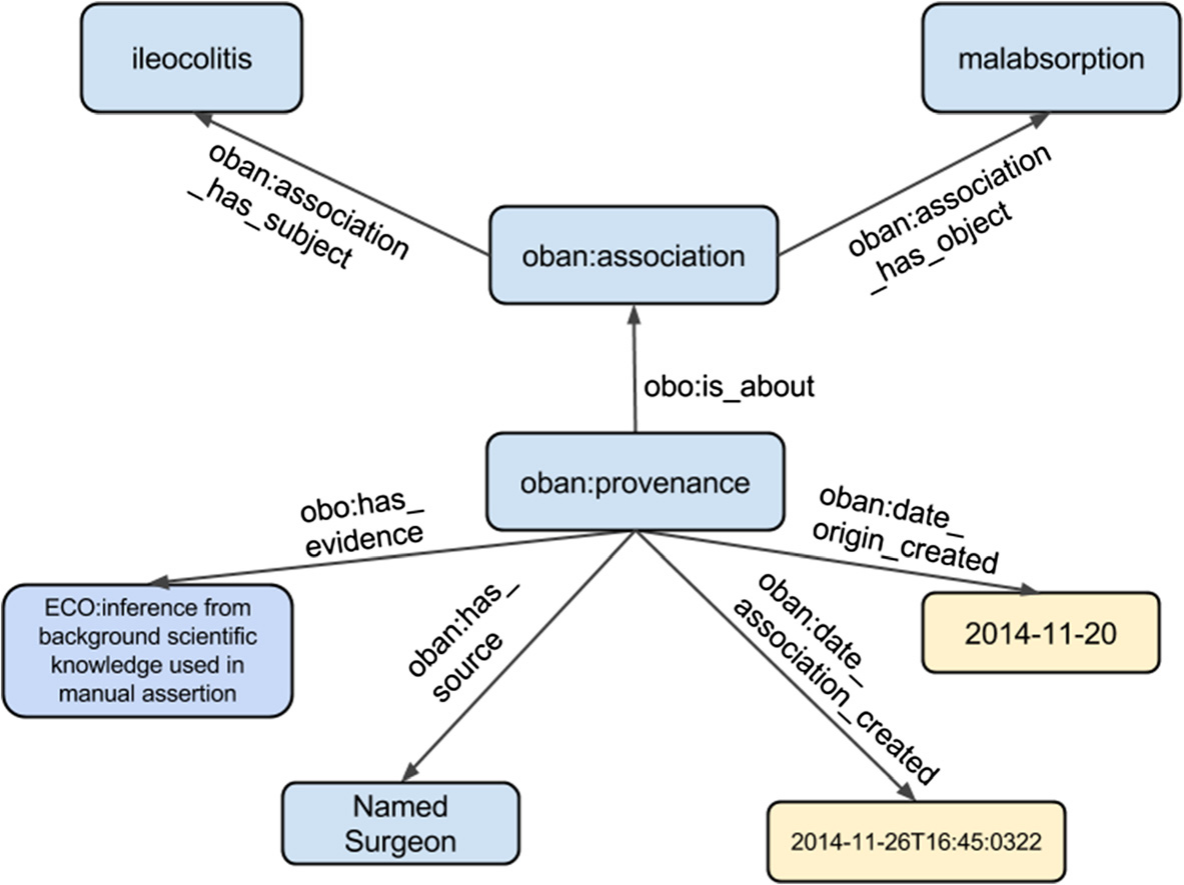
An example of connecting a phenotype (malabsorption) with a disease (ileocolitis) using OBAN. Provenance here is manual curation by a named surgeon (name omitted here)

Figure 4 shows there is an association between the disease *ileocolitis* and the phenotype *malabsorption*, where the provenance is provided via manual curation from a named clinician validating this candidate association as a true positive. In the OWL representation of associations the biological entities are represented using the same URI as the corresponding OWL class rather than represented as individuals – a technique known as punning [32]. Though not crucial, using punning to generate an instance identifier is preferred as it avoids the need to create many new URIs for individuals of the same diseases or phenotypes. In addition, OBAN separates the association between entities from its provenance, i.e., what/who is used in making the assertion. A similar pattern is used in nanopublications [33] and we extend the concept here. Provenance is typed using an extension of the Evidence Code Ontology (ECO) [34] in the OBAN model to allow for extensible triples to be added, such as PubMedID, a curator name or a confidence score and methods for how it was derived as seen in Fig. 3.

## Results

### Extending the ontology with disease axioms

**Fig. 5:**
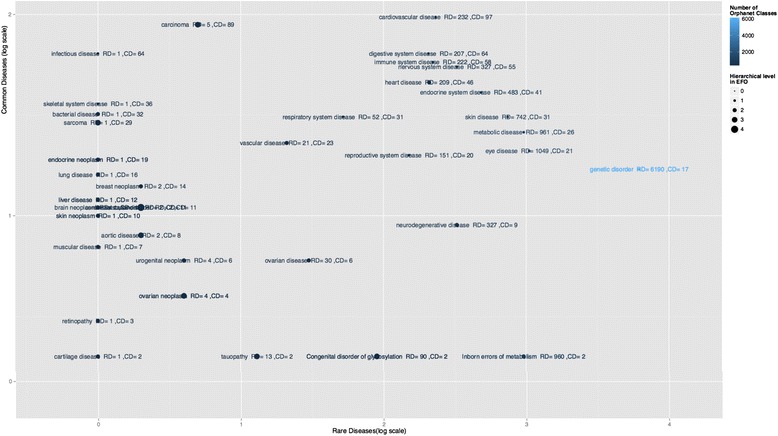
A summary of the rare-to-common associations linking diseases via anatomical system through the *has_disease_location* axiomatization inside EFO. The high-resolution image is downloadable at https://github.com/CTTV/ISMB2015/blob/master/figures/r2c.pdf blob/master/figures/r2c.pdf and provided in supplementary materials

Connections between rare and common diseases in the ontology can be formed through class descriptions where the relation is existentially always true. These relations are hard-coded into EFO by the ontology developers. EFO has been extended to add such descriptions. One such relevant description is in connecting rare and common disease to anatomical organism parts. EFO models this using a simple existential restriction: disease *has_disease_location* some ‘organism part’ where *has_disease_location* is a sub property of the OBO *located_in* object property. EFO version 2.64 (September 2015) contains 1037 such relationships, connecting 5275 diseases to the anatomical areas where they manifest. Figure 5 illustrates the overview of these disease-anatomical parts that cover all anatomical locations, which are shared between rare and common diseases. For the zoomable detailed plot, please consult https://github.com/CTTV/ISMB2015/blob/master/figures/r2c.pdf and Additional file 3.

In another example, the relation between a disease and an abnormality in the biological process is modeled with a class description using the object properties *realized_in*, where a disease is *realized_in* a disease course having an *abnormal* quality that *has_part* a biological process. For example, cancer is axiomatized with (*realized_in* some (‘disease course’ and (*has_part* some ‘cell proliferation’) and (*bearer_of* some abnormal)))). There were 980 disease classes connected to abnormalities in 56 biological processes in EFO version 2.64 that were modeled following the pattern above. EFO is released monthly and available for download at http://www.ebi.ac.uk/efo.

### IBD disease-phenotype associations

Research into Inflammatory Bowel Disease (IBD) is one of the driving use cases for CTTV and as such has been an early focus for this work. The process pipeline in mapping and associating disease-phenotype described in this study is being expanded to cover other CTTV driving use cases in autoimmunity, cancer and has been used for Type 2 diabetes [19]. Over 80 % of all disease and phenotype annotation in resources used in CTTV pipeline were successfully mapped to EFO terms. These resources included ArrayExpress, UniProt, Reactome, GWAS Catalog, ChEBML, and EVA. The results for IBD phenotype mining are available as an OBAN association file at https://sourceforge.net/p/efo/code/HEAD/tree/trunk/src/efoassociations/ibd_2_pheno_associations.owl. The file contains 289 disease-phenotype associations for IBD. After our initial text-mining step, 41.6 % candidate IBD phenotype associations were deemed correct by manual review (precision). Determining the statistics in the error rate for this mining process is challenging as we lack the denominator (false positive) to calculate the false discovery and other error ratios. We identified multiple causes to those disease-phenotype associations that were not manually validated. In some cases, the HP/MP terms that were tagged to the associated disease were non-informative. For example, ‘chronic’, ‘death’, or ‘sudden death’ are valid HP and MP terms. While they were correctly mapped by the mining process, they are not informative enough in establishing the disease-phenotype association and were discarded. In other cases, the inter-annotator agreement among the clinician experts, who specialized in different fields of medicine, varied. A ‘maybe’ or non-verified entry does not signify that the candidate disease-phenotype pair was incorrectly mapped, rather that the experts did not unanimously agree. In those cases, we accepted the association when 2 out of the 3 clinicians agreed.

**Fig. 6:**
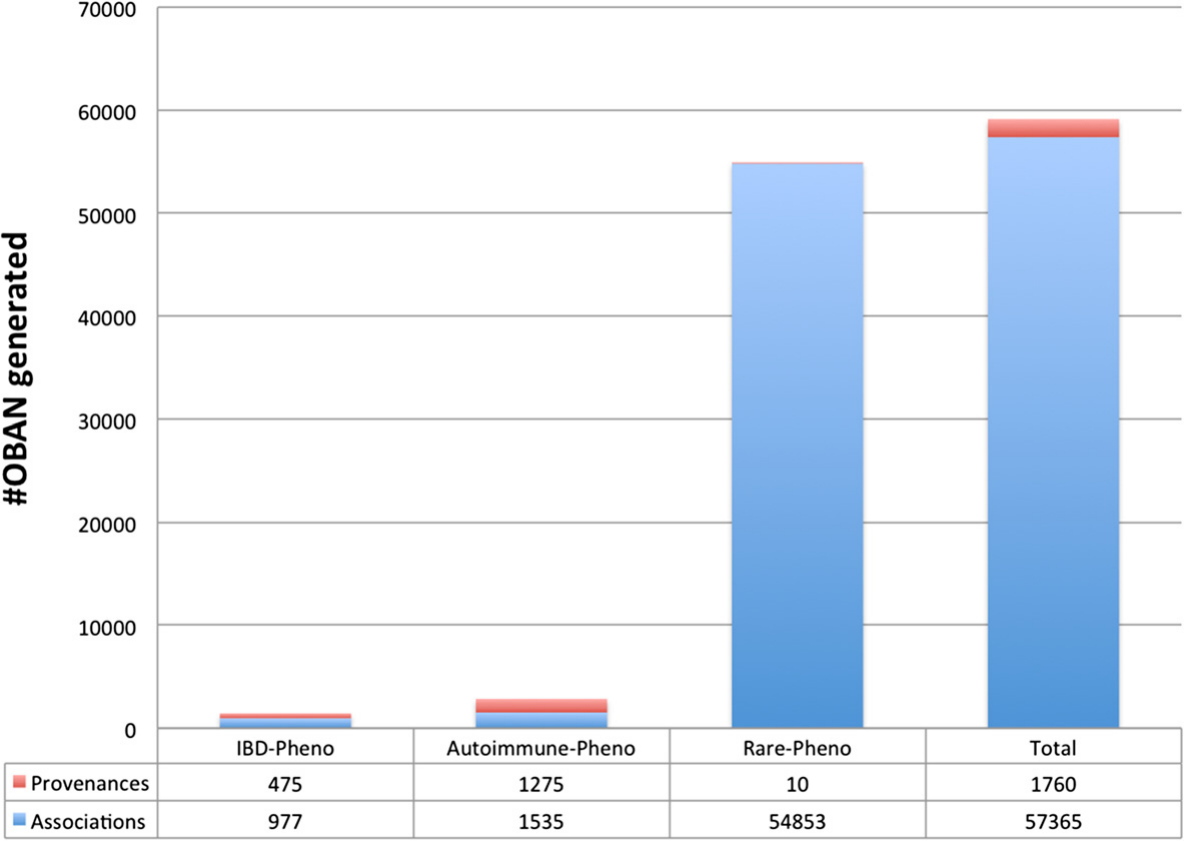
Summary of the number of associations and provenances in each group of diseases in CTTV as of 28th September 2015

To facilitate connection to rare disease, we have extended previous work by the Human Phenotype Ontology and ORDO [22]. We incorporated a subset of the data available from the HP group and extended our disease-phenotype association results with 43,517 individual rare disease-phenotype associations using literature curation and clinician validation (documentation available at http://human-phenotype-ontology.github.io/documentation.html, last accessed 7^th^ October 2015). For instance, connecting colon cancer to Crohn’s disease and to Muir-Torre syndrome (a rare form of colon cancer manifesting in both gastrointestinal and cutaneous systems) provides a connection between disorders which are known to share common phenotypes in cutaneous system such as skin lesions [35]. The complete listing of these rare-to-common diseases via phenotypes are all available in the OBAN model available from http://sourceforge.net/p/efo/code/HEAD/tree/trunk/src/efoassociations/ with the summary statistics provided in Fig. 6. As of September 28^th^ 2015, there were 13,8410 disease-phenotype OBAN associations linked to 1760 provenances in the CTTV knowledge base. By combining the associations to phenotypes from rare diseases, or common diseases we can provide another mechanism for integrating rare and common disease. The current set of associations in this study enables 535 connections between a phenotype and at least one common and at least one rare disease. Such connections can reveal new findings, thereby providing new hypotheses for investigation, or confirm known findings, and providing additional evidence for common mechanisms. Examples from our data include connections for which publications exist, e.g., pruritus which connects both psoriasis and lamella ichthyosis [36], and also those for which publications are harder to find, such as the association between Crohn’s disease and Bannayan-Riley-Ruvalcaba syndrome via cachexia (a syndromic group of symptoms describing the combination of weakness, muscle atrophy, loss of weight, and fatigue).

## Discussion

The understanding and representation of phenotype and disease is both context and domain specific and in both our data mapping and mining experiences, disease and phenotype overlap. Here we operate in the translational research domain specifically to characterize drug targets and to explore phenotypic connections between rare and common disease. Determining what is a disease or phenotype is also confounded by the fact that some of the phenotype terms in HPO are primarily considered diseases, such as Crohn’s disease, and not phenotypes. In EFO we have chosen to omit imported terms in this nature as phenotypes from HP, and represent them as diseases using EFO namespace. EFO also includes an extended range of normal phenotypes, which are out of the scope of HP’s phenotypic abnormalities. Medical language is also multifaceted: a single clinical observation, either a disease or a phenotype, can be represented multiple times with multiple alternative terms and we therefore observe a long tail of annotations which are problematic to map to any ontology.

The literature mining process provides a simple and rapid method to identify ‘candidate’ disease-phenotype associations, which are then curated by expert clinicians and transformed into the OBAN model. The current process could be improved by incorporating aspects of negation detection [37], and applying advanced natural language processing algorithms to the text-mining step. This would also reduce the manual curation effort on the clinician’s end, though we expect that manual review of results will need to remain part of this process. Crowd-sourcing is potentially one approach to help reduce the clinicians’ workload on the manual reviews of the disease-phenotype associations [38]. A phenotypic dissection of disease provides a mechanism to translate the biological complexity to a computational representation to aid in identification and validation of therapeutic targets. The biological subject and object in the OBAN association triples exploit the ontology infrastructure provided in EFO and provide a means to express confidence in annotations using and extending ECO. OBAN provides a robust ontological infrastructure that is complementary to, but more restrictive and detailed than the association representation employed by nanopublication model, which is less ontology-restricted. Nanopublications provide an overarching and generic framework for representing a simple unit of knowledge, but leave the details to each individual publisher. OBAN restricts this model by providing class types and predicates which are to be used to mint new OBAN associations. This is critical when the key aim is immediate data integration, rather than consolidation of many underlying and disparate models for representing a single publication.

The OBAN association model has been successfully applied to represent disease-chromosomal location in the Monarch Initiative [39]. In future work we will include phenotypic frequencies, and disease stage subdivision of phenotypes in collaboration with the clinical community. This will require a revision to the EFO disease hierarchy, which we hope to achieve with the wider community and the Human Disease Ontology in particular.

## Conclusions

Capturing disease-phenotype information with ontology modeling is a multi-step process. Relevant clinical and experimental information benefits from distinguishing between disease and phenotype. We have demonstrated the pipeline for mapping textual information that come from various sources to the corresponding ontology disease or phenotype classes via the mechanism of EFO imports and design patterns. Knowledge of associations may come from various sources: expert’s knowledge, literature mining, or clinical/experimental observations, each with different level of significance. Asserting such knowledge for ontology reasoning may not be done at the class level where the association must always be true, which is often not the case since a disease may have all or some manifestation of different symptoms (*i.e.,* phenotypes). We present an OBAN model that constructs the triple associations exploiting instances of class ‘association’ where traceable provenance of supporting knowledge is asserted per each instance of association. This is a driving mechanism for identifying the connections between rare and common diseases via the shared phenotypes at the Centre for Therapeutic Target Validation. OBAN can also be applied to represent association information other than those of disease-phenotype. Evidence types of disease-target hypotheses such as somatic mutation, genetic association, or affected pathway, once represented with OBAN model, can exploit the full capability of graph computation for ontology reasoning.

### Availability

The EFO and phenotypic associations will be deployed in the CTTV platform, which will be freely available at http://www.targetvalidation.org/ to the community after release in late 2015. EFO is freely available at http://www.ebi.ac.uk/efo/, as are the OBAN associations at https://github.com/EBISPOT/OBAN.

## Additional files

Additional file 1: URLs to the supplementary downloadable result files for text mining results (IBD, Autoimmunity, Skeletal disorders, and Metabolism disorders). (PDF 18 kb) Additional file 2: The list of journals mined for disease-phenotype assocations. (PDF 13 kb) Additional file 3: A zoomable disease-phenotype association by system/organ disease locations. (PDF 9 kb)

## Abbreviations

ATC: Anatomical therapeutic chemical classification
ChEBI: Chemical entities of biological interest
CTTV: Centre for Therapeutic Target Validation
DO: Disease Ontology
ECO: Evidence Code Ontology
EFO: Experimental Factor Ontology
EMBL-EBI: European Molecular Biology Laboratory – European Bioinformatics Institute
EVA: European Variation Archive
GSK: GlaxoSmithKline
HP: Human Phenotype Ontology
IBD: Inflammatory bowel diseases
MedDRA: Medical dictionary for regulatory activities
MeSH: Medical subject heading
MIREOT: Minimum information to reference an external ontology term
MP: Mammalian phenotype ontology
NCIt: National Cancer Institute Thesaurus
OBAN: Ontology for Biomedical AssociatioN
OMIM: Online Mendelian Inheritance in Man
ORDO: Orphanet Rare Disease Ontology
SNP: Single nucleotide polymorphism
SNOMED-CT: Systematized Nomenclature of Medicine – Clinical Terms
URI: Universal resource identifier
WTSI: Wellcome Trust Sanger Institute
