# Supplementary material for: Linking rare and common disease: mapping clinical disease-phenotypes to ontologies in therapeutic target validation
Source: J Biomed Semantics. 2016 Mar 23;7:8. doi: 10.1186/s13326-016-0051-7 (PMC4804633; doi:10.1186/s13326-016-0051-7)
Supplement: Additional file 1: — URLs to the supplementary downloadable result files for text mining results (IBD, Autoimmunity, Skeletal disorders, and Metabolism disorders). (PDF 18 kb) [file 13326_2016_51_MOESM1_ESM.pdf]

Text mining results are available as followed:

IBD:
http://sourceforge.net/p/efo/code/HEAD/tree/trunk/src/efoassociations/ibd.tar.gz

Autoimmunity:
http://sourceforge.net/p/efo/code/HEAD/tree/trunk/src/efoassociations/immune.tar

Skeletal disorders:
http://sourceforge.net/p/efo/code/HEAD/tree/trunk/src/efoassociations/skeletal.tar

Metabolism disorders:
http://sourceforge.net/p/efo/code/HEAD/tree/trunk/src/efoassociations/metabolism.tar
