# Supplementary material for: Linking rare and common disease: mapping clinical disease-phenotypes to ontologies in therapeutic target validation
Source: J Biomed Semantics. 2016 Mar 23;7:8. doi: 10.1186/s13326-016-0051-7 (PMC4804633; doi:10.1186/s13326-016-0051-7)
Supplement: Additional file 2: — The list of journals mined for disease-phenotype assocations. (PDF 13 kb) [file 13326_2016_51_MOESM2_ESM.pdf]

Journals Mined for IBD associations

0193-1857	American Journal of Physiology - Gastrointestinal and Liver Physiology
1108-7471	Annals of Gastroenterology : Quarterly Publication of the Hellenic Society of Gastroenterology
1471-230X	BMC Gastroenterology
1662-0631	Case Reports in Gastroenterology
1178-7023	Clinical and Experimental Gastroenterology
1179-5522	Clinical Medicine Insights. Gastroenterology
1554-7914	Gastroenterology & Hepatology
1687-6121	Gastroenterology Research and Practice
2052-0034	Gastroenterology Report
0017-5749	Gut
2090-8040	International Journal of Inflammation
2090-4398	ISRN Gastroenterology
2090-8695	ISRN inflammation
1476-9255	Journal of Inflammation (London, England)
1178-7031	Journal of Inflammation Research
2154-1280	Journal of Interventional Gastroenterology
1756-283X	Therapeutic Advances in Gastroenterology
1007-9327	World Journal of Gastroenterology : WJG
1948-5190	World Journal of Gastrointestinal Endoscopy
2150-5330	World Journal of Gastrointestinal Pathophysiology
1948-9366	World Journal of Gastrointestinal Surgery
