# Supplementary figures and images for: Linking rare and common disease: mapping clinical disease-phenotypes to ontologies in therapeutic target validation
Source: J Biomed Semantics. 2016 Mar 23;7:8. doi: 10.1186/s13326-016-0051-7 (PMC4804633; doi:10.1186/s13326-016-0051-7)

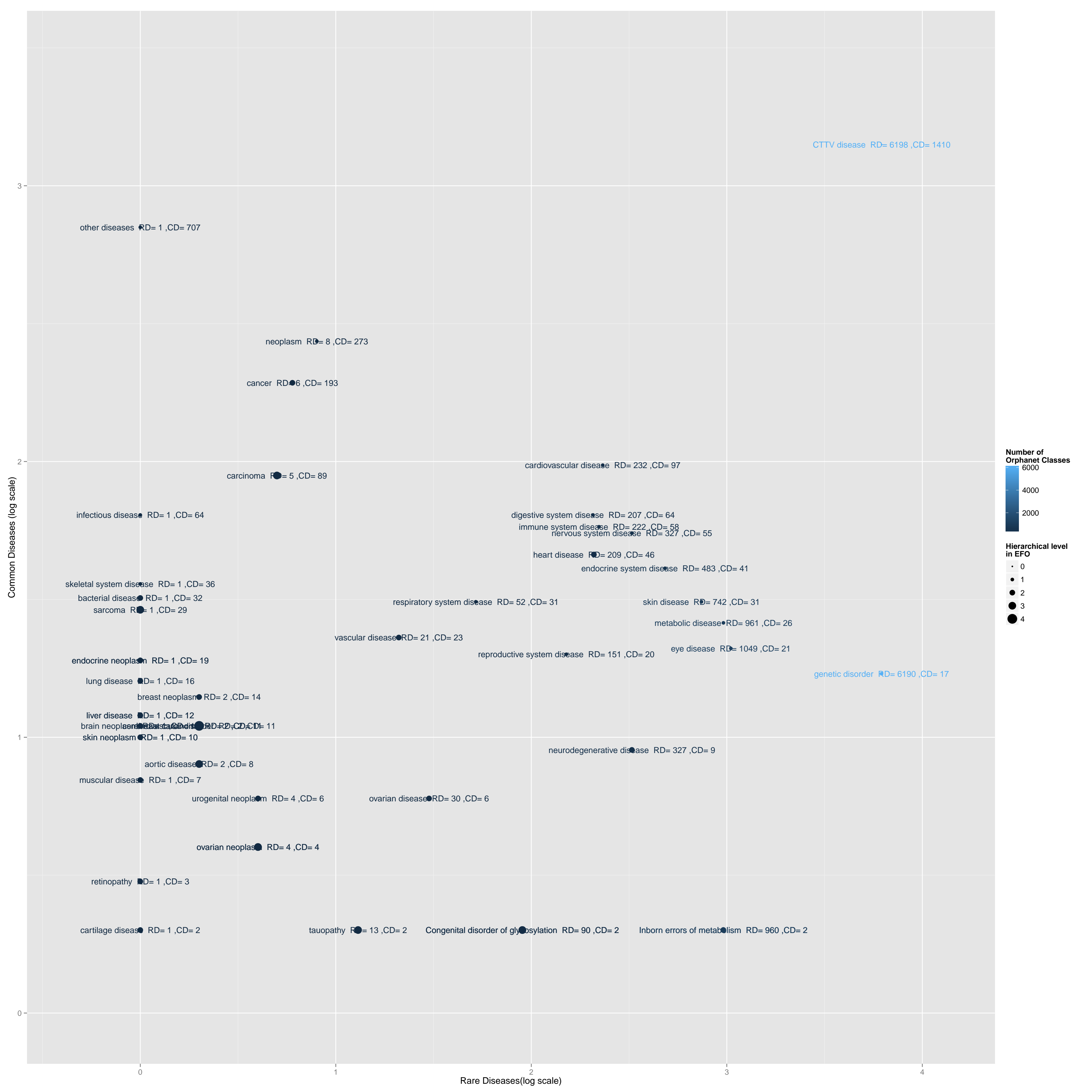

Supplement: Additional file 3: — A zoomable disease-phenotype association by system/organ disease locations. (PDF 9 kb) [file 13326_2016_51_MOESM3_ESM.pdf]
